# Supplementary material for: How the vortex lattice of a superconductor becomes disordered: a study by scanning tunneling spectroscopy
Source: Sci Rep. 2015 Mar 18;5:9244. doi: 10.1038/srep09244 (PMC5378196; doi:10.1038/srep09244)
Supplement: Supplementary Information — How the vortex lattice of a superconductor becomes disordered: a study by scanning tunneling spectroscopy [file srep09244-s1.pdf]

Supplementary information: How the vortex lattice of a superconductor becomes disordered: a study by scanning tunneling spectroscopy

M. Zehetmayer

*Atominstitut, Vienna University of Technology, 1020 Vienna, Austria**

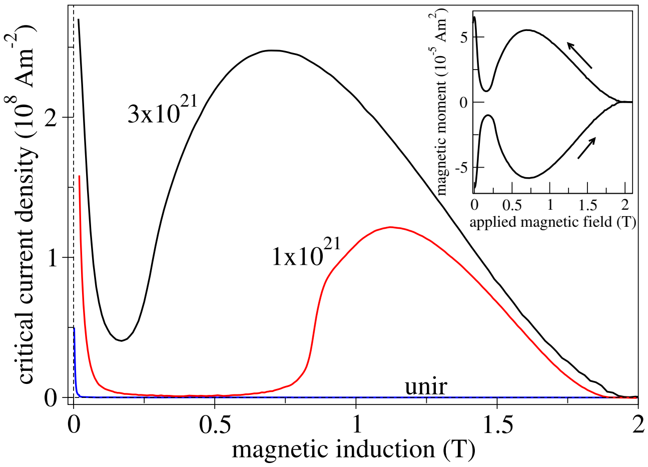

FIG. 1. The critical current density of $NbSe_2$ as a function of magnetic induction at 4.2 K in the unirradiated state (blue line), after neutron irradiation to a fluence (E > 0.1 MeV) of $1 \times 10^{21}\,m^{-2}$ (red line) and $3 \times 10^{21}\,m^{-2}$ (black line). The inset shows the magnetic moment, from which the current was calculated, of the third sample ($3 \times 10^{21}\,m^{-2}$).

I. CRITICAL CURRENT DENSITY

Figure 1 shows the critical current density of single-crystalline $NbSe_2$ in the unirradiated state and after neutron irradiation to a fast neutron ($E > 0.1$ MeV) fluence of $1 \times 10^{21}\,m^{-2}$ and $3 \times 10^{21}\,m^{-2}$. In the irradiated states the critical currents and the vortex lattices were recorded in the same sample, while in the unirradiated state different samples from the same batch, having nearly identical superconducting properties, were employed.

II. FURTHER FLUX-LINE IMAGES

Figure 2 shows the flux-line lattice of an unirradiated sample at 1 T and of a low-irradiated ($1 \times 10^{21}\,m^{-2}$) sample at 0.6 T[1], to which I refer in the main text of the article.

III. SCANNING TUNNELING SPECTROSCOPY

A. Measurements

Scanning tunneling spectroscopy was carried out at 4.2 K employing a commercial microscope with a liquid helium cryostat including a 7 T superconducting magnet. Using silver

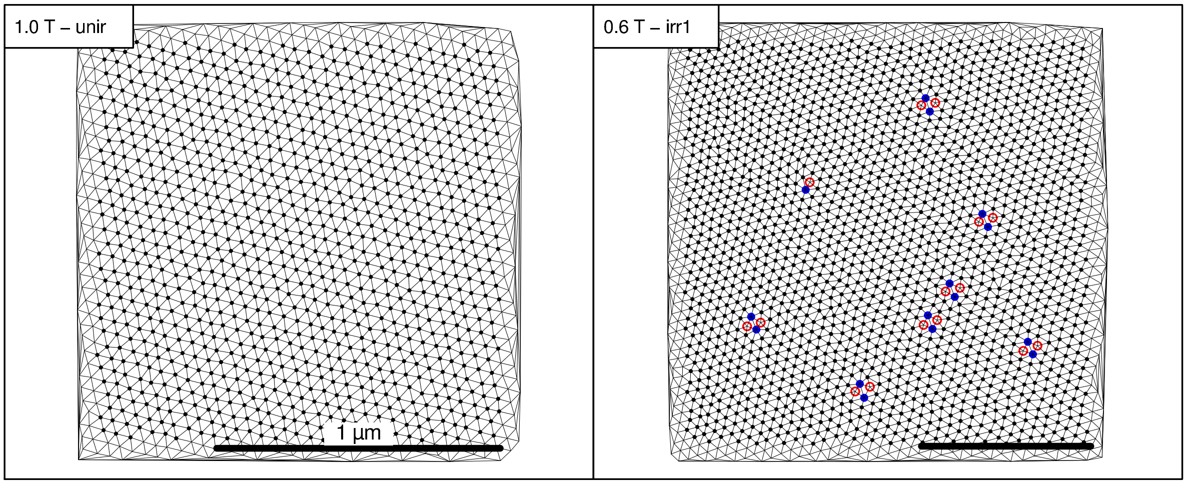

FIG. 2. The vortex lattice of an unirradiated sample at 1 T and of a low-irradiated sample at 0.6 T.

paste, I glued the sample on a piezo scanner that has a range of $33 \times 33\,\mu\text{m}^2$ parallel and $2.5\,\mu$m perpendicular to the sample surface. The measurements were carried out with a PtIr tip. The tunneling current (I) was set to 400 pA and the voltage (V) usually to 1.2 meV, which is well below the superconducting gap edge of some 2 meV. A lock-in technique was employed to obtain the derivative dI / dV, which is proportional to the density of states. Dependent on the applied field, a step-size of 1-2 nm was chosen, allowing me to record a flux-line lattice with up to 3000 vortices within about 3-5 days. Measurements started several hours after applying the magnetic field, so that a further relaxation of the vortex distribution played no significant role.

B. Image processing

Figure 3 shows a part of a density of states map resulting from a measurement of a sample at 0.25 T. The circular dark spots reveal the positions of the vortices. For further processing, the background was corrected line-wise and then a Gaussian filter with a radius close to the radius of the vortex cores employed. These procedures usually removed most of the noise so that the vortex centers could be determined by automatically searching for the local maxima. Finally, I carefully compared the resulting vortex positions with the original image and, if necessary, amended them by hand.

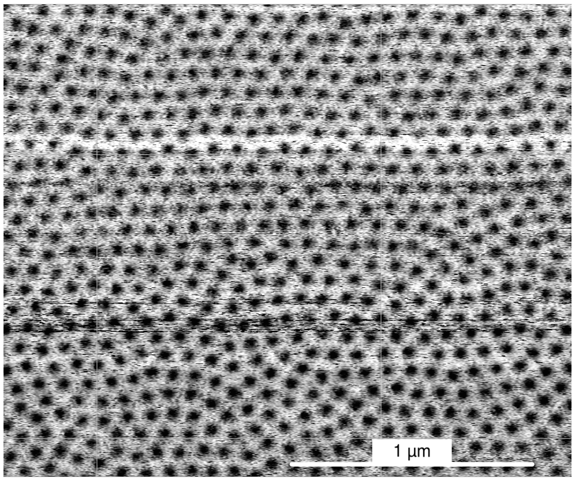

FIG. 3. The density of states map of an irradiated sample at 0.25 T. The dark circular spots show the vortex cores.

IV. CORRELATION FUNCTIONS

The envelopes of the correlation functions have been used to identify different states of matter by comparing them with a power law, namely $r^{-\eta}$, and an exponential law, $e^{-\xi/r}$, with η and ξ (the correlation length) as fit parameters[2–5] (see[6] for a review).

For the Bragg glass, theory predicts that the orientational correlation function remains constant and the translational function decays as a power law with $\eta < 1/3$. These presumptions are fulfilled in the unirradiated sample; for instance, at 1 T the orientational correlation is virtually constant (the power-law fit would lead to $\eta \simeq 0.003$) and the translational correlation changes as $r^{-0.038}$, i.e. $\eta < 1/3$.

The disordered (vortex glass) state is expected to follow an exponential law with a relatively short correlation length as was indeed found for our disordered sample. The exponential fits at 0.5 T, shown in figure 4 of the article, reveal correlation lengths of 1.5 - 2 a_0. Unlike theory, this behavior occurs merely at not too large distances, while a flatter curve is observed at larger distances, indicating some remaining long-range correlation. This long-range correlation may be an aftermath of the ordered state that is passed through when increasing the magnetic field from 0 (or a negative value) to the measuring point.

Regarding the hexatic state, the orientational order is expected to be still (quasi) long-range, indicated by the power law with $\eta < 1/4$, while the translational correlation should be short-range, indicated by the exponential law. I found a power-law decay of the orientational

order near the onset of the second peak, with $\eta \simeq 0.06$ for both 0.18 and 0.20 T and $\eta \simeq 0.15$ for 0.25 T, that is $\eta < 1/4$ in all cases. A power law works also best for the translational correlation but with a much faster decay of $\eta > 1/3$ indicating a bad long-range order. In particular $\eta \simeq 0.35$ was found for 0.18 T, $\eta \simeq 0.53$ for 0.2 T, and $\eta \simeq 0.55$ for 0.25 T. Exponential fits work again only for small ranges and result in correlation lengths of 3-4 a_0. Accordingly, the behavior of the correlation functions is indicative of the hexatic state though the predicted courses are not exactly met.

---

* zehetm@ati.ac.at

[1] Hecher, J., Zehetmayer, M. & Weber, H. W. How the macroscopic current correlates with the microscopic flux-line distribution in a type-II superconductor: an experimental study. *Supercond. Sci. Technol.* **27**, 075004 (2014).

[2] Kosterlitz, J. M. & Thouless, D. J. Ordering, metastability and phase transitions in two-dimensional systems. *J. Phys. C* **6**, 1181 (1973).

[3] Nelson, D. R. & Halperin, B. I. Dislocation-mediated melting in two dimensions. *Phys. Rev. B* **19**, 2457–2484 (1979).

[4] Halperin, B. I. & Nelson, D. R. Theory of two-dimensional melting. *Phys. Rev. Lett.* **41**, 121–124 (1978).

[5] Young, A. P. Melting and the vector coulomb gas in two dimensions. *Phys. Rev. B* **19**, 1855–1866 (1979).

[6] von Grünberg, H. H., Keim, P. & Maret, G. *Soft Matter Volume 3, edited by G. Gompper, M. Schick* Ch. 2 (WILEY-VCH, Weinheim, 2007).
